# Comprehensive analysis of fibroblast activation protein expression across 23 tumor indications: insights for biomarker development in cancer immunotherapies

**DOI:** 10.3389/fimmu.2024.1352615

**Published:** 2024-03-15

**Authors:** Sebastian Dziadek, Anton Kraxner, Wei-Yi Cheng, Tai-Hsien Ou Yang, Mike Flores, Noah Theiss, Tsu-Shuen Tsao, Emilia Andersson, Suzana Vega Harring, Ann-Marie E. Bröske, Maurizio Ceppi, Volker Teichgräber, Jehad Charo

**Affiliations:** ^1^Roche Pharma Research and Early Development, Oncology, Roche Innovation Center Basel, F. Hoffmann-La Roche Ltd., Basel, Switzerland; ^2^Roche Pharma Research and Early Development, Data and Analytics, Roche Translational & Clinical Research Center, F. Hoffmann-La Roche Ltd, Little Falls, NJ, United States; ^3^Roche Tissue Diagnostics, Tucson, AZ, United States; ^4^Roche Pharma Research and Early Development, Oncology, Roche Innovation Center Munich, Roche Diagnostics GmbH, Penzberg, Germany; ^5^Roche Pharma Research and Early Development, Oncology, Roche Innovation Center Zurich, Roche Glycart AG, Schlieren, Switzerland

**Keywords:** fibroblast activation protein, immunohistochemistry, mRNA expression, immunotherapy, clinical outcomes

## Abstract

**Introduction:**

Fibroblast activation protein (FAP) is predominantly upregulated in various tumor microenvironments and scarcely expressed in normal tissues.

**Methods:**

We analyzed FAP across 1216 tissue samples covering 23 tumor types and 70 subtypes.

**Results:**

Elevated FAP levels were notable in breast, pancreatic, esophageal, and lung cancers. Using immunohistochemistry and RNAseq, a correlation between FAP gene and protein expression was found. Evaluating FAP’s clinical significance, we assessed 29 cohorts from 12 clinical trials, including both mono and combination therapies with the PD-L1 inhibitor atezolizumab and chemotherapy. A trend links higher FAP expression to poorer prognosis, particularly in RCC, across both treatment arms. However, four cohorts showed improved survival with high FAP, while in four others, FAP had no apparent survival impact.

**Conclusions:**

Our results emphasize FAP’s multifaceted role in therapy response, suggesting its potential as a cancer immunotherapy biomarker.

## Introduction

Recent breakthroughs in cancer immunotherapy, especially the adoption of anti-PD-1/PD-L1 treatments for multiple cancers, have transformed cancer care. The tumor microenvironment (TME) – a multifaceted network of non-malignant cells surrounding tumors – is crucial in determining clinical outcomes and immunotherapy responses (1). Recent studies indicate that fibroblast activation protein (FAP) on cancer-associated fibroblasts (CAFs) can promote tumor inflammation and suppress innate and adaptive anti-tumor immunity (2). FAP, also termed “seprase,” is a 170 kDa type II membrane-bound serine protease (3–5). FAP expression, noted in various cancers, often hints at prognosis. In hepatocellular carcinoma, FAP rises under tumor microenvironment hypoxia, aligning with adverse outcomes (6). Osteosarcoma shows heightened FAP linked to tumor size and stage (7). Importantly, in prostate cancer, high FAP mRNA levels correlate with aggressive disease and decreased survival (8).

The prevailing literature largely points to an inverse relationship between FAP expression and clinical prognosis. However, some studies present conflicting evidence. For instance, Park et al. found that a decreased presence of FAP-positive CAFs correlated with diminished survival rates in pancreatic ductal carcinoma patients (9). Ariga et al. highlighted FAP expression as an independent predictor for extended overall and disease-free survival in breast cancer cases (10).

In this study, we analyzed FAP expression in 1,216 tumor samples across 23 tumor types and 70 subclassifications. Using both immunohistochemistry (IHC) and RNA sequencing, we identified a relationship between FAP protein and its mRNA levels. Additionally, we studied the impact of FAP expression on clinical outcomes in clinical trials investigating the anti-PD-L1 agent atezolizumab, either as mono- or combination therapy as well as chemotherapy regimens.

## Materials and methods

### Patients and samples

Tumor samples as well as a normal multi-tissue array (30 tissues, BC8 array, SuperBioChips, South Korea) were sourced from commercial tissue banks (AMSBIO, Pantomics) or from deidentified patients enrolled in Roche non-randomized and randomized independent multicenter open-label phase I dose escalation studies and a phase II multicenter open-label basket clinical trials who provided informed consent. Data were used according to internal processes and guidelines and were analyzed for all treated patients that donated biopsy, regardless of intention-to-treat status. These samples included primary tumors, metastases, and lymph node metastases, procured either via core needle biopsies or tumor resections. **Table 1** outlines the details of the early-phase studies sponsored by Roche from which these tumor samples were obtained.

**Table 1:** Roche-supported, early- and late-phase studies.

Study ID	Description
BP39365 NCT03063762	An Open-Label, Multi-Center, Randomized, Dose-Escalation, Phase 1b Study to Evaluate Safety, Pharmacokinetics and Therapeutic Activity of RO6874281 in Combination With Atezolizumab ± Bevacizumab in Patients With Unresectable Advanced and/or Metastatic Renal Cell Carcinoma
BP29842 NCT02627274	An Open-Label, Multicenter, Dose-Escalation, Phase Ia/Ib Study to Evaluate Safety, Pharmacokinetics, and Therapeutic Activity of RO6874281, an Immunocytokine Consisting of Interleukin 2 Variant (IL-2v) Targeting Fibroblast Activation Protein-α (FAP), as a Single Agent (Part A) or in Combination With Trastuzumab or Cetuximab (Part B or C)
BP40234 NCT03386721	An Open-Label, Multicenter, Phase II Study to Evaluate the Therapeutic Activity of Simlukafusp Alfa (RO6874281), an Immunocytokine, Consisting of Interleukin-2 Variant (IL-2v) Targeting Fibroblast Activation Protein-α (FAP), in Combination With Atezolizumab (Anti-PD-L1), Administered Intravenously, in Participants With Advanced and/or Metastatic Solid Tumors
GO28625 NCT01846416	A Study of Atezolizumab in Participants With Programmed Death-Ligand 1 (PD-L1) Positive Locally Advanced or Metastatic Non-Small Cell Lung Cancer (NSCLC) [FIR]
GO28753 NCT01903993	A Randomized Phase 2 Study of Atezolizumab (an Engineered Anti-PDL1 Antibody) Compared With Docetaxel in Participants With Locally Advanced or Metastatic Non-Small Cell Lung Cancer Who Have Failed Platinum Therapy - “POPLAR”
GO28754 NCT02031458	A Study of Atezolizumab in Participants With Programmed Death - Ligand 1 (PD-L1) Positive Locally Advanced or Metastatic Non-Small Cell Lung Cancer (BIRCH)
GO28915 NCT02008227	A Study of Atezolizumab Compared With Docetaxel in Participants With Locally Advanced or Metastatic Non-Small Cell Lung Cancer Who Have Failed Platinum-Containing Therapy (OAK)
GO29436 NCT02366143	A Study of Atezolizumab in Combination With Carboplatin Plus (+) Paclitaxel With or Without Bevacizumab Compared With Carboplatin+Paclitaxel+Bevacizumab in Participants With Stage IV Non-Squamous Non-Small Cell Lung Cancer (NSCLC) (IMpower150)
GO29437 NCT02367794	A Study of Atezolizumab in Combination With Carboplatin + Paclitaxel or Carboplatin + Nab-Paclitaxel Compared With Carboplatin + Nab-Paclitaxel in Participants With Stage IV Squamous Non-Small Cell Lung Cancer (NSCLC) [IMpower131]
GO30081 NCT02763579	A Study of Carboplatin Plus Etoposide With or Without Atezolizumab in Participants With Untreated Extensive-Stage (ES) Small Cell Lung Cancer (SCLC) (IMpower133)
GO29293 NCT02108652	A Study of Atezolizumab in Participants With Locally Advanced or Metastatic Urothelial Bladder Cancer (Cohort 2) (IMvigor 210)
GO29294 NCT02302807	A Study of Atezolizumab Compared With Chemotherapy in Participants With Locally Advanced or Metastatic Urothelial Bladder Cancer [IMvigor211]
WO29074 NCT01984242	A Study of Atezolizumab (an Engineered Anti-Programmed Death-Ligand 1 [PD-L1] Antibody) as Monotherapy or in Combination With Bevacizumab (Avastin®) Compared to Sunitinib (Sutent®) in Participants With Untreated Advanced Renal Cell Carcinoma (IMmotion150)
WO29637 NCT02420821	A Study of Atezolizumab in Combination With Bevacizumab Versus Sunitinib in Participants With Untreated Advanced Renal Cell Carcinoma (RCC) (IMmotion151)
WO29522 NCT02425891	A Study of Atezolizumab in Combination With Nab-Paclitaxel Compared With Placebo With Nab-Paclitaxel for Participants With Previously Untreated Metastatic Triple-Negative Breast Cancer (IMpassion130)
GO28625 NCT01846416	A Study of Atezolizumab in Participants With Programmed Death-Ligand 1 (PD-L1) Positive Locally Advanced or Metastatic Non-Small Cell Lung Cancer (NSCLC) [FIR]

### Immunohistochemistry

Consecutive 4-μm sections from formalin-fixed paraffin-embedded (FFPE) tumor tissues were prepared for immunohistochemistry (IHC) analysis. The Ventana FAP (SP325) Robust Prototype Assay (RPA) by Ventana Medical Systems Inc. (Tucson, AZ, USA) was used for the staining process. This assay, which uses a rabbit monoclonal antibody, clone SP325, obtained from Spring Biosciences (Pleasanton, CA, USA), was designed to detect Fibroblast Activated Protein (FAP) in FFPE samples. The staining was carried out using the OptiView DAB IHC Detection^TM^ kit on a VENTANA BenchMark ULTRA instrument. To evaluate the reactivity of the secondary antibody and detection chemistry, an immunoglobulin-matched rabbit monoclonal antibody (VMSI, Catalog No. 790-4795) was employed as a negative control. The staining intensity was scored manually on a semi-quantitative scale ranging from 0 (negative) to 3 (or “3+”) by a certified anatomic pathologist. The percentage of cells stained positively, covering both normal stroma and neoplastic cells, was recorded for each intensity level. An H-score from 0-300 was calculated by combining the stromal and tumor cell staining (FAP-intensity score). The stroma-tumor H-score incorporated components from both normal stromal and tumor staining intensities along with the percentage of positively stained cells.

### Bulk RNA sequencing

RNA and DNA were simultaneously extracted from formalin-fixed, paraffin-embedded (FFPE) samples for Bulk RNA Sequencing. The process involved the generation of Illumina TruSeq RNA Access Sequencing Libraries, adhering to the protocol ID (LAB_13_3256) as outlined by Q2S/Expression Analysis (US). The extraction utilized core-needle tumor biopsies. For this purpose, eight FFPE sections, each measuring 4-5 microns and cumulatively amounting to approximately 40 microns, were required. The mRNA fraction was selectively enriched within these libraries by employing a set of biotinylated oligonucleotides targeting coding regions of the genome. The library preparation protocol necessitated a minimum RNA input of 100 ng. Samples exhibiting a %DV200 value below 30 were deemed unsuitable for this analytical method. The sequencing was executed on an Illumina platform, employing a 50bp paired-end sequencing approach. The objective was to achieve a sequencing depth of 40 million reads per sample.

In the subsequent phase, the generated Fastq files were transferred to Roche for processing through the Biokit pipeline. The conversion of BCL to FASTQ files was accomplished using Illumina’s bcl2fastq converter, version 2.17.1.14, which facilitated base calling (source: Illumina Downloads). To quantify gene expression levels, the paired-end RNASeq reads were aligned to the human genome reference (hg38) utilizing the STAR aligner, version 2.5.2a. This process adhered to the default mapping parameters, including the ‘reverse’ option under the stranded setting. The quantification of reads was conducted via the featureCounts software; it aggregated the read counts mapped to all Ensembl transcript variants of each gene into a consolidated figure. These counts were then normalized to represent counts per million (cpm), providing a standardized measure of gene expression.

We performed RNA sequencing (RNASeq) analysis on a series of samples, including FIR, POPLAR, BIRCH, OAK, and various immunotherapy trial data sets (IMPOWER150, IMPOWER131, IMPOWER133, IMVIGOR210, IMVIGOR211, IMMOTION150, IMMOTION151, IMPASSION130). The RNASeq reads from these samples were meticulously aligned to the human genome reference GRCh38 using the GSNAP algorithm (11, 12). Subsequent to alignment, the reads corresponding to exons in each RefSeq gene were quantified. This quantification was executed using the GenomicAlignments package from the R/Bioconductor suite, ensuring a robust and reliable analysis. The resultant read counts were then normalized to counts per million (cpm) to facilitate comparative analysis across samples.

### Statistical analyses

Spearman’s rank-order correlation coefficient (ρ) was applied to evaluate the strength and direction of monotonic relationships between two ordinal or continuous variables, particularly when the data did not conform to normality or linearity assumptions. Prior to computing ρ, data ranking was performed, with tie adjustments made as necessary. Spearman’s ρ values span from -1 to 1: values approaching 1 indicate a strong positive correlation, those nearing -1 suggest a strong negative correlation, and values around 0 imply an absence of monotonic correlation. The significance of these correlations was determined using a p-value threshold of less than 0.05. These analyses were conducted using Python and the SciPy package.

In this research, the hazard ratio (HR) was utilized to quantify the relative risk of an event’s occurrence between two groups. The HR offers an estimate of instantaneous risk throughout the study period and is crucial in survival or time-to-event analyses. An HR greater than 1 indicates an elevated risk in the treatment or exposure group relative to the control group, whereas an HR less than 1 denotes a decreased risk. The Cox proportional hazards model facilitated the computation of adjusted hazard ratios, accounting for potential confounders. Additionally, 95% confidence intervals for the HRs were calculated to assess the precision of these estimates. All survival analyses were executed using Python with the lifelines package.

## Results

### Expression of FAP in normal and tumor specimens

**Figure 1:**
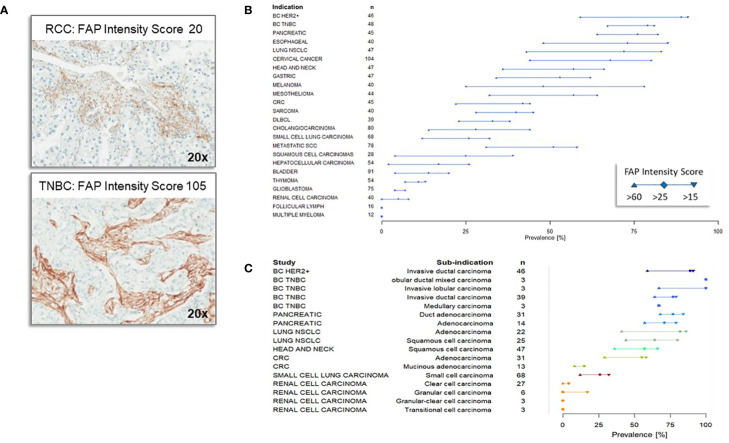
Immunohistochemistry of Fibroblast Activation Protein (FAP) expression across different tumor types and subtypes **(A)** Representative immunohistochemistry staining of Fibroblast Activation Protein (FAP). The top panel illustrates a renal cell carcinoma (RCC) specimen with a FAP intensity score of 20, indicating low FAP expression, while the bottom panel shows a triple negative breast cancer specimen with an FAP intensity score of 105, indicating high FAP expression. **(B)** Fibroblast Activation Protein (FAP) Expression Across Different Tumor Types. FAP expression levels (with cutoffs of >15, >25 and >60 intensity score) across various tumor types are displayed in descending order based on their relative abundance of expression, as quantified by the FAP intensity score at the cutoff of >25. The number of samples analyzed for each tumor type is denoted by ‘n.’ The majority of the specimens were obtained from primary tumors. **(C)** Expression of Fibroblast Activation Protein (FAP) across different subtypes of tumors within specific indications. The comprehensive analysis included a total of 70 distinct subtypes, with only selected representative types displayed in the figure. Subtypes of tumors of the same indication are displayed in the same color.

Using a mirco-array spanning a range of 30 normal tissue types, no or very low levels of FAP staining were detected except for organs with areas of remodeling tissue (proliferating endometrium, placenta). FAP staining within tumor specimens primarily localized in the stromal component adjacent to tumor cells. Heterogeneity in FAP distribution was evident, presenting as thick, moderate, or thin strands, even within the same tumor subtypes. While some cases had equivalent FAP area coverage, the distribution variations highlighted the complex relationship between heterogeneity and tumor morphology. **Figure 1A** demonstrates FAP expression in tumor tissues, consistently staining the reactive stroma around tumor areas. The top panel depicts a FAP intensity score of 20 in an RCC sample, while the bottom one shows a score of 105 in a triple negative breast cancer (TNBC) case. Using the FAP intensity score criteria, we categorized 1216 samples across tumor types with cut-off values of >15, >25, and >60. High FAP expression was most prevalent in breast, pancreatic, esophageal, and lung cancers, with the least in renal cell carcinoma, follicular lymphoma and myeloma (**Figure 1B**). Among solid cancer subtypes, invasive ductal, mixed, and lobular breast carcinomas had the highest FAP expression. In contrast, granular and transitional granular clear cell kidney cancers had the least (**Figure 1C**), emphasizing their rarity among patients.

**Figure 2:**
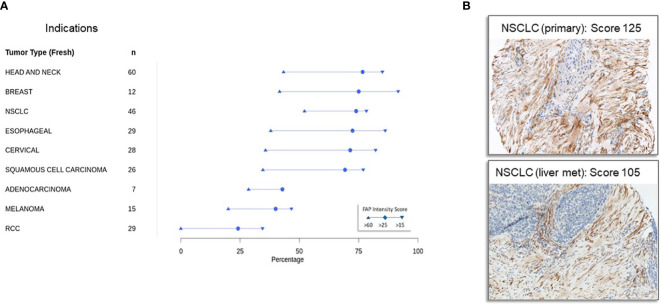
Immunohistochemical detection of fibroblast activation protein expression in tumor samples in patients with advanced cancers from early-phase clinical trials **(A)** expression of fibroblast activation protein in advanced cancers from early-phase clinical trials: Immunohistochemistry was conducted on tumor specimens obtained from a cohort of patients participating in early phase, Roche-sponsored clinical trials. The staining protocol employed here mirrors that outlined in **Figure 1** and is detailed in the Materials and Methods section. Notably, a significant proportion of these specimens originated from patients with metastatic disease who had progressed beyond primary therapy, making the study cohort particularly relevant for assessing FAP expression in advanced stages of disease. **(B)** Representative immunohistochemistry staining of FAP in specimens derived from a non-small cell cancer (NSCLC) primary tumor with a FAP intensity score of 125 (top panel) and a NSCLC metastatic liver lesion with an FAP intensity score of 105 (bottom panel).

**Figure 2A** shows FAP immunohistochemistry results from samples sourced from patients in early-phase Roche-supported clinical trials. In contrast to **Figure 1**, which focuses on primary tumors, **Figure 2A** highlights metastatic disease samples. Many of these metastatic samples originated from patients in second or subsequent lines of treatment trials, making them particularly insightful for assessing FAP expression in advanced tumors. This is crucial given that the TME and treatment response can differ markedly between primary and advanced tumors.

Our analysis discerned variations in FAP expression between these tumor stages. Nonetheless, a consistent pattern emerged: head and neck, breast, lung, and esophageal tumors exhibited the highest FAP expression, whereas renal cell carcinoma (RCC) demonstrated low levels. This suggests that FAP might play a divergent role in the pathophysiology of certain cancers, offering potential avenues for developing targeted treatments. **Figure 2B** demonstrates that FAP expression is consistently high in non-small cell cancer primary tumors (top panel, FAP intensity score of 125) and metastatic lesions (bottom panel, NSCLC liver metastasis, FAP intensity score of 105).

### Correlation between FAP IHC and mRNA expression

**Figure 3:**
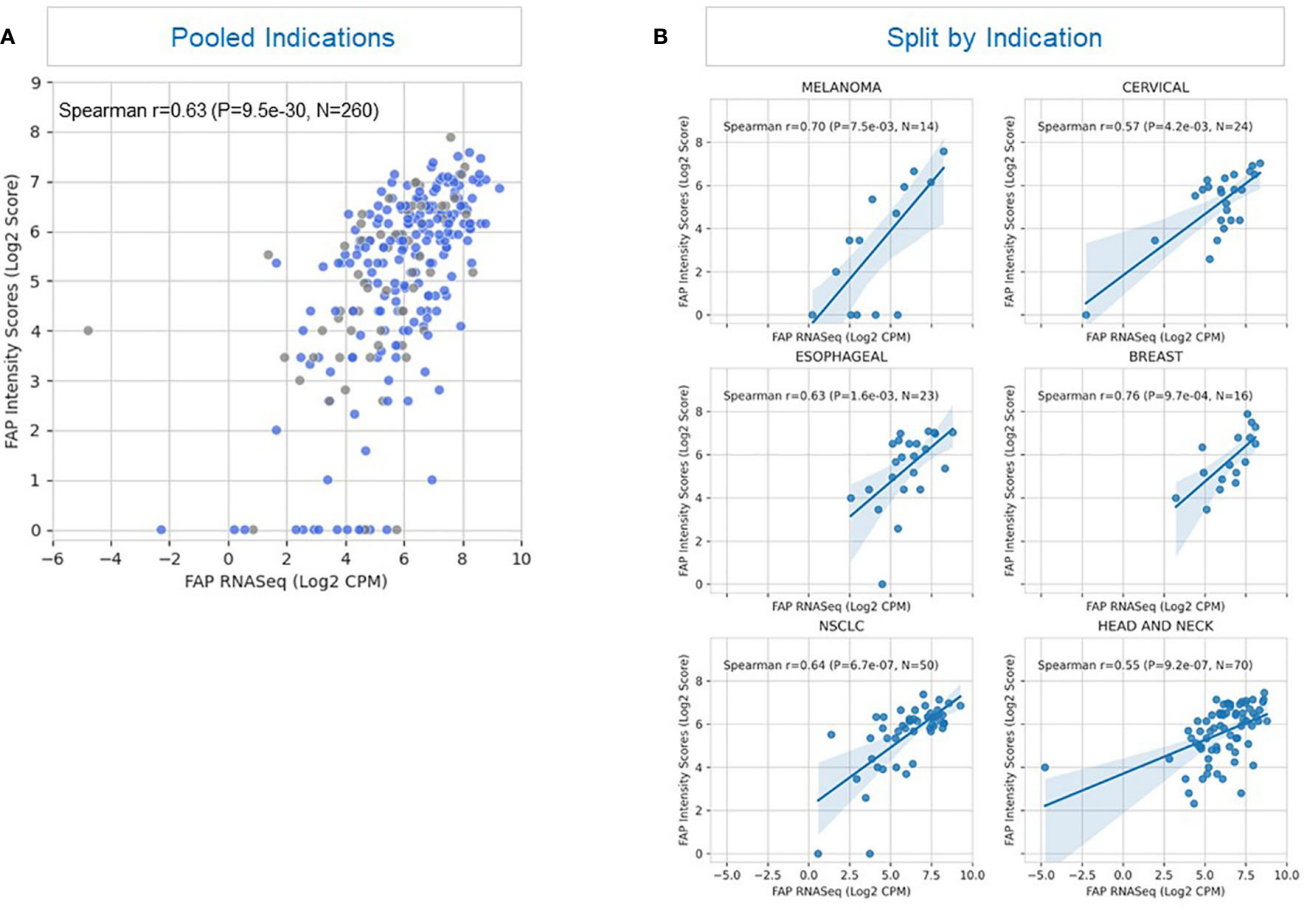
Correlation between FAP expression detected by immunohistochemistry and RNAseq. **(A)** The scatterplot illustrates the correlation between FAP (Fibroblast Activation Protein) expression levels in a tissue sample as detected by two different methods: immunohistochemistry and RNAseq. Blue represents fresh samples (samples provided within 28 days after tissue acquisition; n=195) whereas grey depicts archived samples (samples provided after more than 28 days after tissue acquisition; n=65). The plot depicts cumulative results of all samples irrespective of tumor type. Each point on the plot represents an individual sample (n= 260) from a heterogeneous tumor population. The x-axis displays FAP expression values obtained through RNAseq analysis, represented as transformed counts per million (log2 CPM). The y-axis displays FAP expression levels determined by immunohistochemistry, quantified as a semi-quantitative score based on staining intensity and distribution, and represented as log2-transformed FAP intensity score (log2 Score). A positive correlation (Spearman’s correlation coefficient ρ = 0.63, p < 0.001) between the two methods is observed, suggesting that FAP expression levels assessed by immunohistochemistry correspond closely to those measured through RNAseq analysis. This correlation indicates the reliability of either immunohistochemistry or RNAseq as methods for evaluating FAP expression in this tissue context. **(B)** This figure displays Spearman’s correlation coefficient (ρ) representing the relationship between FAP (Fibroblast Activation Protein) expression levels measured by immunohistochemistry (y-axis) and RNAseq data (x-axis) in six illustrative tumor types: breast cancer, esophageal cancer, NSCLC (Non-Small Cell Lung Cancer), cervical cancer, HNSCC (Head and Neck Squamous Cell Carcinoma), and melanoma. The y-axis represents FAP expression levels determined by immunohistochemistry, measured on a semi-quantitative scale, and represented as log2-transformed FAP intensity score (log2 Score). The x-axis shows FAP expression levels obtained through RNAseq analysis, measured as log2-transformed counts per million (log2 CPM). The Spearman’s correlation coefficients (ρ) range between 0.55 and 0.76, indicating varying degrees of correlation between the two measurement methods across different tumor types. Notably, the highest correlation (ρ = 0.76) is observed in breast cancer samples, suggesting a strong concordance between immunohistochemistry and RNAseq data for FAP expression in breast cancer. In contrast, the lowest correlations (r = 0.55 and 0.57 are seen in non-small cell lung cancer and cervical cancer, indicating greater variability or discordance in FAP expression measurements in these tumor types.

We validated our immunohistochemistry (IHC) results by establishing the correlation between protein levels and FAP mRNA expression, as determined by RNAseq in fresh and archived tumor samples (**Figure 3A**). The correlation coefficient had an overall r value of 0.63 for all specimens (n=260) signifying an association between protein and mRNA expression.

Further analysis revealed a correlation between FAP IHC and mRNA expression for most tumor types with the greatest correlation noted for breast cancer and melanoma, as illustrated in **Figure 3B**. Renal cell carcinoma (not shown) had a lower level of association which could potentially be attributed to the overall diminished levels of FAP expression in RCC tumors combined with the limited availability of samples with matched results for IHC and RNAseq. These findings provide insights into the reliability of immunohistochemistry as a method for assessing FAP expression in different tumor contexts, with implications for its use in research and clinical settings.

### Correlation between FAP expression and clinical outcomes in atezolizumab trials

Simlukafusp alfa (FAP-IL2v), an immunocytokine designed to specifically bind to FAP via an antibody portion with an IL2 portion modified to be biased toward IL-2Rβγ while abolishing its binding to IL-2Rα, has demonstrated promising results. In an orthotopic, syngeneic mouse model of pancreatic cancer, FAP-IL-2v exhibited synergistic effects when combined with a murine anti-PD-L1 antibody, significantly improving the survival of mice compared to monotherapy with the anti-PD-L1 antibody (13). In an early-phase human trial involving FAP-IL-2v in combination with the anti-PD-L1 drug atezolizumab for patients with metastatic or recurrent cervical cancer, a favorable safety profile and significant anti-tumor activity were observed compared to approved PD-1 inhibitors (14).

Simlukafusp alfa with FAP targeting and atezolizumab (atezo) represent two distinct approaches in cancer therapy, each with unique mechanisms of action and therapeutic targets. Simlukafusp alfa, a fusion protein, combines fibroblast activation protein (FAP) targeting with interleukin-2 variant (IL2v), focusing primarily on modifying the tumor microenvironment. FAP, expressed in cancer-associated fibroblasts within the tumor stroma, is targeted to reduce the stroma’s support for tumor growth, with the IL2v component stimulating immune responses for a potentially enhanced therapeutic effect. In contrast, atezolizumab operates through a broader mechanism, functioning as an anti-PD-L1 antibody that blocks the PD-L1 protein on tumor cells. This action inhibits the PD-1/PD-L1 immune checkpoint pathway, reactivating T cells to attack a wide range of tumor types. While simlukafusp alfa with FAP targeting aims at stromal modulation and localized immune stimulation, atezolizumab’s impact is more generalized, focusing on reinvigorating the immune system against tumors by targeting a key immune checkpoint. These differences underscore the diverse strategies and potential impacts each has in the realm of cancer treatment.

**Figure 4:**
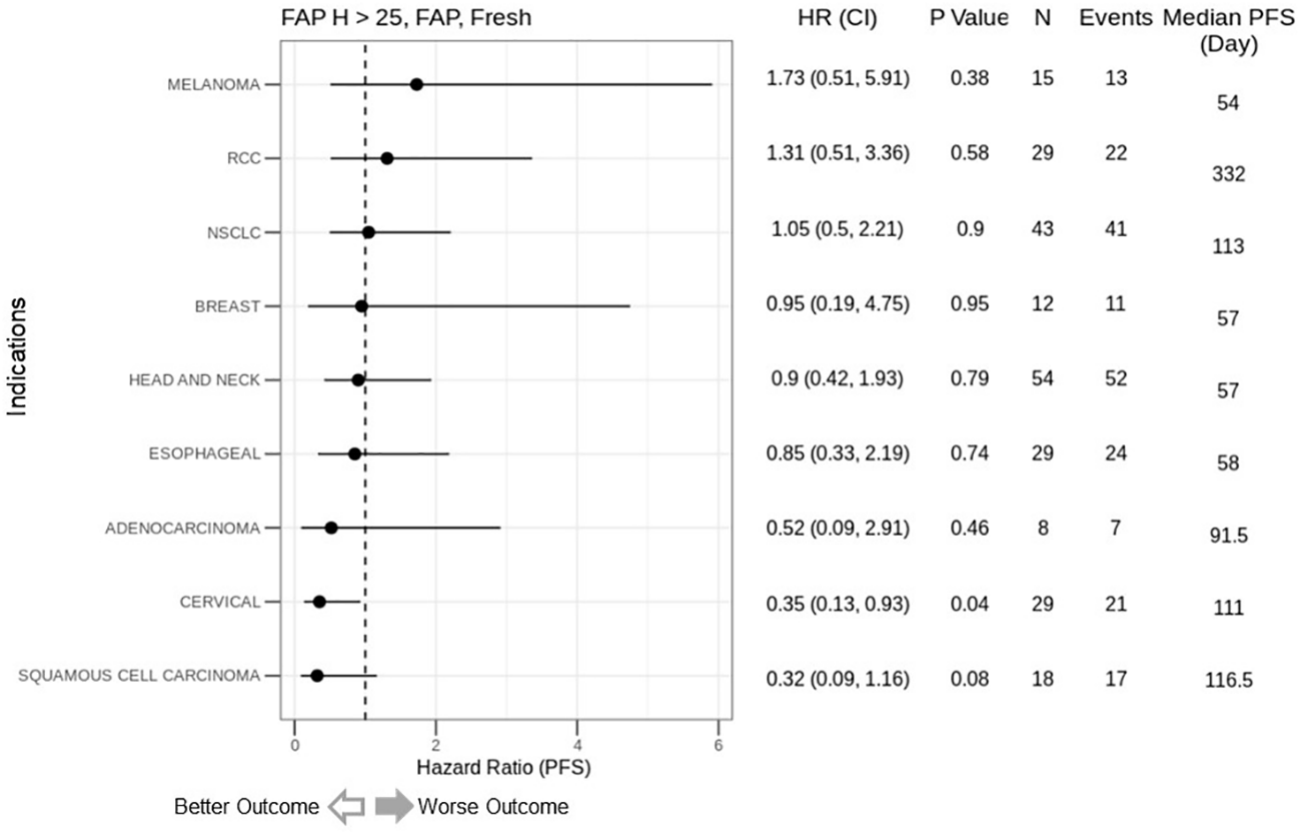
Correlation of FAP intensity score (>25) with Progression-Free Survival (PFS) in cancer immunotherapy trials. This figure presents forest plots depicting the hazard ratios (HR) for PFS based on FAP intensity scores from simlukafusp alfa studies (refer to **Table 1**), categorized by cancer type. Data are expressed as HR with 95% confidence intervals (CI) in parentheses. Notably, higher FAP expression correlates with a numerically inferior HR in melanoma and renal cell carcinoma (RCC) cohorts, contrasting with a significantly improved HR in cervical cancer patients. The analysis encompasses 237 patients from three early-phase, Roche-sponsored clinical trials evaluating simlukafusp alfa/FAP-IL2v in combination with checkpoint inhibitors (Atezolizumab or Pembrolizumab) across various solid tumor types. FAP expression levels were determined via immunohistochemistry (IHC), with a positivity threshold set at an H-score greater than 25. The impact on PFS varied across tumor types, showing a non-significant trend toward better outcomes in Squamous Cell Carcinoma and a significant improvement in Cervical Cancer, while indicating a non-significant negative trend in Melanoma. HR, Hazard Ratio; CI, Confidence Interval; N, Number of Patients.

We explored the correlation between FAP expression and clinical outcomes in simlukafusp alfa trials, spanning four tumor indications. Utilizing IHC to determine FAP expression, we assessed nine distinct tumor types, linking FAP expression with progression-free survival (PFS). Among these, adenocarcinoma, cervical cancer, and squamous cell carcinoma exhibited improved outcomes with heightened FAP expression. Conversely, melanoma and RCC showed worse outcomes. Notably, outcomes in head and neck, NSCLC, and esophageal carcinomas appeared to be unaffected by FAP expression (**Figure 4**).

**Figure 5:**
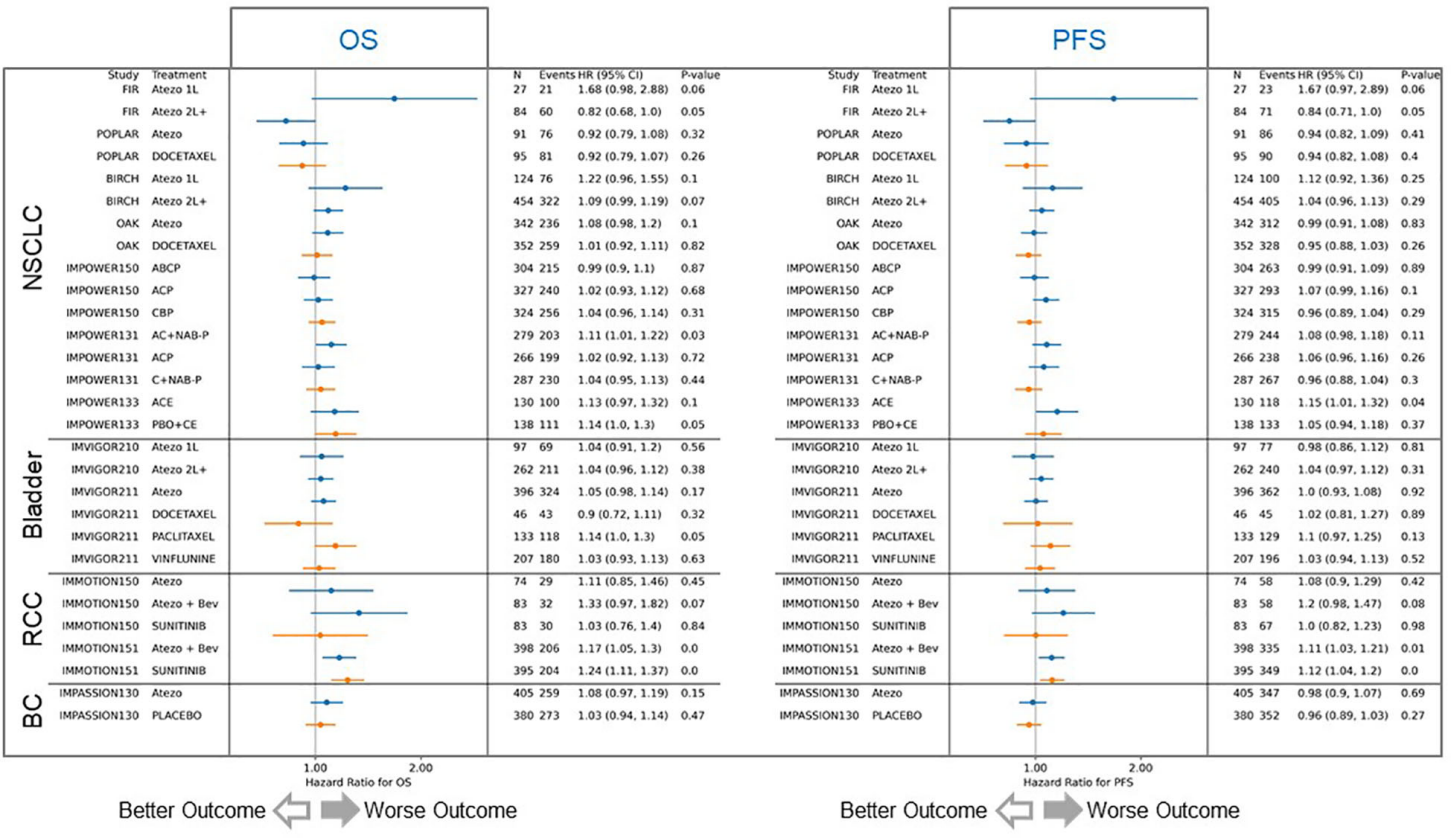
Correlation of FAP expression with overall and progression-free survival outcomes. The analysis included 29 patient cohorts from 12 trials, with over 6000 samples. Among 18 atezolizumab-treated cohorts (blue lines), 10 displayed worse prognosis with elevated FAP expression, 5 showed no significant effect, and 2 had slightly better prognosis. In contrast, among 11 chemotherapy cohorts (orange lines), only 3 exhibited worse progression-free survival (PFS) and overall survival (OS) with high FAP expression. Atezo, atezolizumab; Bev, bevacizumab; ACP, atezolizumab plus carboplatin plus paclitaxel; BCP, bevacizumab plus carboplatin plus paclitaxel; ABCP, atezolizumab plus bevacizumab plus carboplatin plus paclitaxel; AC+NAB-P, atezolizumab plus carboplatin plus nab-paclitaxel; C+NAB-P, carboplatin plus nab-paclitaxel; ACE, atezolizumab plus carboplatin plus etoposide; PBO+CE, placebo plus carboplatin plus etoposide.

We subsequently investigated the relationship between FAP expression and clinical outcomes in atezolizumab trials, both as monotherapy and in combination with anti-angiogenesis or chemotherapy, across four different tumor indications. Our analysis encompassed 29 patient cohorts from 12 trials, utilizing more than 6000 samples. RNAseq was the method used to evaluate the correlation between FAP expression and clinical outcome. Among the 18 cohorts treated with atezolizumab, 10 displayed a worse prognosis associated with elevated FAP expression, while 5 showed no significant effect, and 2 exhibited marginally better prognosis (**Figure 5**, blue lines). Of the 11 chemotherapy cohorts, only 3 displayed worse progression-free survival (PFS) and overall survival (OS) associated with high FAP expression (**Figure 5**, orange lines). The data reveals a significant correlation between higher FAP expression and (A) decreased overall survival and (B) reduced progression-free survival, suggesting a potential prognostic value of FAP expression in this cohort. It is important to note that this effect is not universal and may vary between different patient cohorts, highlighting the complex interplay between FAP expression and survival outcomes.

## Discussion

The utilization of the immune system to combat cancer, often referred to as cancer immunotherapy, has undergone significant evolution since its inception. Initially, global research efforts were focused on identifying unique tumor antigens that could serve as targets for cytotoxic T cells, aiming to induce tumor destruction (15). Unfortunately, this approach yielded limited clinical benefits (16, 17). The advent of checkpoint blockade inhibitors marked a turning point, significantly improving the clinical outlook for various cancer types (18). Nevertheless, a substantial fraction of patients remain unresponsive to checkpoint blockade therapy. This realization has prompted a shift in our understanding toward the tumor microenvironment (TME) and the exploration of strategies to modulate it in conjunction with cancer immunotherapy.

Our study has unveiled varying degrees of FAP expression across different tumor types and subtypes. Furthermore, our analyses and previous reports have highlighted the limited or absent expression of FAP in normal, non-malignant tissues, contrasting with its abundance in tumor stroma and pericytes of tumor neovasculature (19, 20). The establishment of a single and robust method across varying tumor and specimen types presents a pivotal advancement in the systematic assessment of FAP (Fibroblast Activation Protein) rule in TME. A unified approach not only standardizes the measurement, reducing potential variations and discrepancies associated with multiple techniques, but also streamlines the comparative analysis across diverse malignancies. This comprehensive method would enable a more consistent correlation between FAP expression and clinical outcomes, thereby providing a clearer understanding of FAP’s prognostic significance. Such a standardization can drive the optimization of therapeutic strategies targeting FAP, facilitating better patient stratification and potentially improving treatment outcomes across the oncologic spectrum.

In murine models, the pivotal role of FAP-positive CAFs in promoting tumor growth by suppressing antitumor immunity has been established (21). Targeting CAFs in models of Lewis lung cancer (LLC1) or B16F1 melanoma has been shown to enhance antitumor CD8+ T cell responses (22). Moreover, depleting FAP+ fibroblasts in pancreatic ductal adenocarcinoma mice resulted in tumor shrinkage and the generation of robust antitumor immunity (23). Similarly, in a murine model of pancreatic ductal carcinoma, the depletion of FAP+ stromal cells potentiated the antitumor effects of anti-CTLA4 and anti-PD-L1 therapy (24). Consequently, FAP-targeted immunotherapies hold the potential to dismantle the local immunosuppressive environment imposed by CAFs.

Importantly, our study represents the first attempt to correlate FAP expression with outcomes in more than 15 early to phase III clinical trials involving both immunotherapeutic and non-immunotherapeutic anticancer agents. While our empirical findings indicate that FAP expression is often associated with inferior clinical outcomes in most indications, exceptions were noted where FAP expression was associated with a positive impact or had no discernible effect. Consequently, our results underscore the need to validate FAP as a potential biomarker across various tumor types, clinical histories (treatment-naïve versus prior therapies), and treatment modalities. Such validation efforts are crucial for better stratification and identification of patient populations that stand to benefit the most from FAP-targeted therapies, either in combination with or as an adjunct to checkpoint inhibitor therapy.

Interestingly, our preliminary findings, as illustrated in **Figures 2**, **4**, indicate a distinct efficacy profile for the FAP-targeting immunotherapy, simlukafusp alfa. Specifically, this therapy appears less effective in conditions with lower FAP expression, such as melanoma and renal cell carcinoma (RCC), while showing increased effectiveness in conditions with high FAP expression, notably cervical cancer. This pattern suggests that targeting FAP in immunotherapy could potentially counteract its suppressive effects in cases where FAP expression is elevated. However, to validate these observations and underlying hypotheses, further investigation through randomized controlled trials, comparing simlukafusp alfa with non-FAP-targeted therapies, is essential. The intricate relationships between various stromal cell types, including Cancer-Associated Fibroblasts (CAFs) and immune cells, necessitate simultaneous evaluation for a holistic understanding. Focusing solely on FAP (Fibroblast Activation Protein) without considering the broader cellular context may not provide a comprehensive insight into TME dynamics. The FAP content, while crucial, is only one piece of the puzzle. Equally important is the spatial organization and proximity of these cells to tumor cells and immune cells. This spatial relationship can profoundly influence tumor progression and the overall immune response. Notably, while transcriptomic analyses offer valuable molecular insights, they fall short in revealing these spatial intricacies. Immunohistochemistry (IHC) is a superior tool in this regard, enabling a detailed visualization of cellular interactions and spatial distributions that can be pivotal in shaping therapeutic strategies and predicting disease progression.

## Data availability statement

Due to Roche company policy and patient privacy reasons access to individual patient level data is restricted. Qualified researchers may request access to individual patient-level data through the clinical study data request platform (RRID:SCR_018080, https://vivli.org/). Further details on Roche's criteria for eligible studies are available here (https://vivli.org/ourmember/roche/). For further details on Roche's Global Policy on the Sharing of Clinical Information and how to request access to related clinical study documents, see here (https://www.roche.com/innovation/process/clinical-trials/data-sharing/).

## Ethics statement

All procedures were conducted in accordance with the Helsinki declaration and following ethics approval. Patient participation in clinical trials and specimen acquisition was performed with informed consent. Patient tumor samples included in this study were only used if appropriate informed consent was available. Data were used according to internal processes and guidelines and were analyzed for all treated patients that donated biopsy, regardless of Intention-to-treat status.

## Author contributions

SD: Conceptualization, Methodology, Supervision, Writing – original draft, Writing – review & editing. AK: Conceptualization, Investigation, Methodology, Writing – original draft, Writing – review & editing. W-YC: Conceptualization, Data curation, Formal analysis, Methodology, Visualization, Writing – review & editing. T-HOY: Conceptualization, Data curation, Formal analysis, Methodology, Visualization, Writing – review & editing. MF: Data curation, Formal analysis, Methodology, Writing – review & editing. NT: Conceptualization, Data curation, Formal analysis, Methodology, Writing – review & editing. T-ST: Data curation, Formal analysis, Methodology, Writing – review & editing. EA: Conceptualization, Writing – review & editing. SV: Conceptualization, Writing – review & editing. A-MB: Conceptualization, Writing – review & editing. MC: Conceptualization, Writing – review & editing. VT: Conceptualization, Writing – review & editing. JC: Conceptualization, Methodology, Supervision, Writing – review & editing.
